# Standardised dual-flow cytometry workflows for quantitative assessment of mitochondrial superoxide and mitochondrial mass in live cancer cells and primary multiple myeloma cells

**DOI:** 10.3389/fcell.2026.1854542

**Published:** 2026-07-02

**Authors:** Eugenia Giglio, Martina Giuseffi, Marzia Sichetti, Marisabel Mecca

**Affiliations:** Laboratory of Preclinical and Translational Research, Centro di Riferimento Oncologico della Basilicata (IRCCS-CROB), Rionero in Vulture, Italy

**Keywords:** flow cytometry, live cancer cells, mitochondrial mass, mitochondrial superoxide, MitoSOX, MitoTracker, ROS

## Abstract

Mitochondrial metabolism plays a critical role in carcinogenesis and cancer progression. Quantitative assessment of mitochondrial function in live cells remains technically challenging because existing biochemical assays lack single-cell resolution, and microscopy-based approaches are limited in throughput and quantitative reproducibility. Here we describe a robust and reproducible standardised dual-flow cytometry protocol for simultaneous quantitative assessment of mitochondrial superoxide production and mitochondrial mass in live cancer cells and primary patient-derived multiple myeloma plasma cells using MitoSOX Green and MitoTracker Red. The protocol provides a step-by-step workflow comprising preparation of cultured cancer cells or isolation of primary CD138^+^ plasma cells, optimised probe staining, viability discrimination, standardised flow cytometry acquisition and gating, and quantitative fluorescence normalisation. Compared with conventional mitochondrial assays requiring cell lysis or imaging-based analysis, this approach enables high-throughput, quantitative mitochondrial profiling at single-cell resolution in heterogeneous populations while preserving cellular integrity. The procedure incorporates defined staining conditions, instrument calibration guidance, quality-control criteria and normalisation strategies to improve reproducibility across experiments and laboratories. The workflow yields robust fluorescence measurements with low technical variability and enables discrimination of mitochondrial oxidative activity relative to mitochondrial content, facilitating analysis of mitochondrial dysfunction, oxidative stress responses and treatment-induced mitochondrial perturbations. The method is compatible with multiparametric flow cytometry and can be adapted to diverse cell types and experimental systems. The complete protocol requires ∼6–8 h for cultured cells or 8–12 h when primary cell isolation is included and can be implemented by researchers with standard cell culture and flow cytometry expertise.

## 1 Introduction

In recent years, an increasing number of studies have focused on the crucial role of mitochondrial metabolism in tumorigenesis and cancer progression. Mitochondria, the predominant source of energy production in most eukaryotic cells, play critical roles in various vital processes, including adenosine triphosphate (ATP) production, metabolism, biosynthesis, apoptosis and redox signalling, and their dysregulation contributes to tumour initiation, progression and therapy resistance (Henze and Martin, 2003; Porporato et al., 2018; Cadenas and Davies, 2000). Mitochondria are also the major endogenous source of reactive oxygen species (ROS), which, together with reducing and oxidizing species in the organelle (NAD^+^/NADH, FAD/FADH_2_, NADP^+^/NADPH, glutathione/glutathione disulfide), determine mitochondrial metabolic activity and overall fitness.

ROS, generated by electron leakage from the electron transport chain or by monoamine oxidase amine deamination in the outer mitochondrial membrane (Cadenas and Davies, 2000) can lead to lipid peroxidation and protein and nucleic acid damage and are associated with cancer and other diseases (Kumar et al., 2015; Hoye et al., 2008; Zhu et al., 2016). Indeed, ROS also play a significant role in carcinogenesis mechanisms, as tumour cells often exhibit abnormally high levels of ROS generated by deregulated metabolism (Cheung and Vousden, 2022). Increased intracellular oxidative stress, associated with ROS production, often promotes cellular damage and somatic mutations, leading to cancerous transformation. In particular, in cancer cells, ROS generation is often increased due to exposure to a hypoxic microenvironment, where low oxygen levels increase mitochondrial dysfunction and promote anaerobic metabolism, further increasing oxidative stress and contributing to tumour progression and resistance to therapy (Chen et al., 2023). Quantitative assessment of mitochondrial function in living cells remains technically challenging because conventional biochemical approaches lack compartmental resolution, imaging-based methods are limited in throughput and quantitative reproducibility, and the redox state of different compartments may change during mitochondrial isolation (Liao et al., 2020). Fluorescent mitochondrial probes such as MitoSOX and MitoTracker enable non-invasive evaluation of mitochondrial superoxide production and mitochondrial mass in living cells.

**FIGURE 1:**
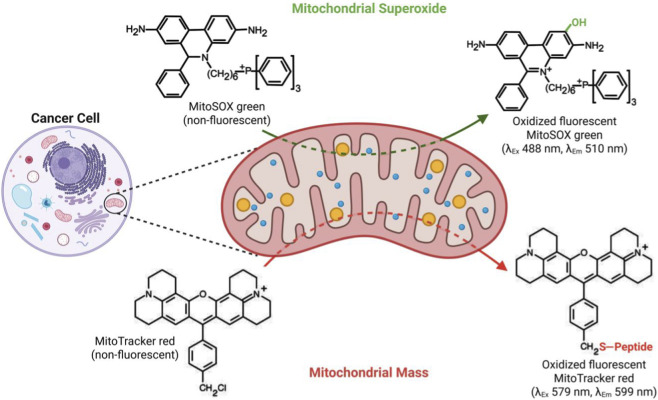
Detection of mitochondrial superoxide and mitochondrial mass using MitoSOX Green and MitoTracker Red. Schematic representation of mitochondrial probe–based analysis in cancer cells. MitoSOX Green selectively accumulates in mitochondria and becomes fluorescent upon oxidation by mitochondrial superoxide (λ_ex 488 nm, λ_em 510 nm), enabling quantification of mitochondrial reactive oxygen species. MitoTracker Red accumulates in mitochondria in a membrane potential–dependent manner and becomes covalently retained following oxidation (λ_ex 579 nm, λ_em 599 nm), providing a measure of mitochondrial mass. Together, these probes enable simultaneous assessment of mitochondrial functional status by flow cytometry. Created in BioRender by Mecca, M (2026).

Specifically, MitoSOX is oxidised by superoxide (O_2_^−^) into ethidium, a positively charged molecule. The positively charged ethidium is retained within the mitochondria and can then intercalate into the mitochondrial DNA. This intercalation of ethidium into mitochondrial DNA leads to a significant increase in the fluorescent signal, which can be quantitatively measured and is directly proportional to the total mitochondrial ROS level (Kauffman et al., 2016). When oxidised by mitochondrial superoxide, MitoSOX produces either bright green or red fluorescence, depending on the fluorogenic dye used (Figure 1). In this specific protocol, the green dye MitoSOX is employed because of its ability to permeate living cells and rapidly and selectively target mitochondria. Once inside the mitochondria, the MitoSOX Green (MSG) reagent is oxidised by superoxide, which emits bright green fluorescence (Pitsillidou et al., 2023). This probe is preferentially oxidised by superoxide, not by other ROS (such as hydrogen peroxide (H_2_O_2_) or hydroxyl radicals (OH)) or reactive nitrogen species (RNS). Additionally, the oxidation of MitoSOX Green is inhibited by superoxide dismutase and superoxide scavengers, further confirming its selectivity for superoxide detection. Rotenone, a known complex I inhibitor of the mitochondrial respiratory chain, was used as a positive control. Rotenone’s inhibition of electron transport leads to an accumulation of electrons and subsequent generation of mitochondrial superoxide radicals and others ROS (Radad et al., 2006; Cheng et al., 2023). This rapid increase in mitochondrial superoxide production makes rotenone an effective positive control for assessing oxidative stress and mitochondrial dysfunction.

MitoTracker probes are used to measure mitochondrial mass by providing a fluorescent signal, making them valuable tools in cell biology for visualizing mitochondria (Chazotte, 2011). These probes consist of a lipophilic cationic dye capable of passively diffusing across the plasma membrane and accumulating within mitochondria in living cells (Figure 1). The accumulation occurs because the cationic dye is attracted to and concentrated within the mitochondrial matrix due to the highly negative membrane potential across the inner mitochondrial membrane (Perry et al., 2011). A key advantage of MitoTracker probes is their ability to remain in the mitochondria even after the loss of membrane potential during fixation. This retention is due to the formation of a covalent bond with the thiol group of a cysteine residue within the mitochondria (Bak et al., 2017). The structure of MitoTracker varies depending on the specific dye used, with red, green, dark red, and orange variants available. In this protocol, MitoTracker Red (MTR) is employed. The dye generally has a planar aromatic structure with two positively charged nitrogen atoms, which allows it to accumulate in active mitochondria in a membrane potential-dependent manner (Figure 1). Once inside the mitochondria, the dye binds to mitochondrial proteins, causing them to fluoresce. The fluorescence intensity is proportional to the dye concentration inside the mitochondria, providing insights into mitochondrial mass and morphology (Neikirk et al., 2023).

Although several studies have previously employed mitochondrial fluorescent probes in flow cytometry assays, most available approaches focus on isolated mitochondrial parameters, lack standardised acquisition and gating criteria, or are optimised primarily for immortalised cell lines. The present workflow has been developed to address the limitations of operator dependence, quantitative reproducibility and mitochondrial isolation. It introduces a harmonised dual-probe strategy that enables the simultaneous quantification of mitochondrial superoxide production and mitochondrial mass at single-cell resolution via flow cytometry. This is achieved under standardised and reproducible conditions that are applicable to both immortalised cancer cells and primary patient-derived samples.

### 1.1 Comparison to other methods

Over the years, various biochemical methods have been developed to assess the cellular redox status, each with distinct advantages and limitations.

One of the common methods used to assess cellular oxidative status is the GSH/GSSG ratio. Reduced glutathione (GSH) is one of the most important scavengers of ROS and can be considered a critical marker of oxidative stress. The ratio of oxidized glutathione (GSSG) to GSH is commonly used to assess the redox state of a cell (Zitka et al., 2012). However, these methods measure GSH/GSSG levels across all cellular compartments without resolving compartment-specific distributions and require cell lysis, preventing analysis of live cells and heterogeneous populations.

Mitochondrial isolation–based approaches enable direct biochemical analysis of mitochondrial function, including respiratory activity, membrane potential and ROS production, in purified organelles obtained by differential centrifugation or density gradient separation and subsequently analysed using enzymatic or respirometry-based assays (Will et al., 2006). Although these methods provide detailed mechanistic insight under controlled experimental conditions, they require extensive sample manipulation that may introduce artefacts and compromise mitochondrial integrity, membrane potential, and redox state. In addition, they are time-consuming, require large numbers of cells and are not suitable for single-cell or heterogeneous population analyses.

In contrast, the use of membrane-permeable non-invasive fluorescence dyes enables real-time visualization of the mitochondrial redox state in living cells, overcoming many of the artefacts associated with sample processing (Liao et al., 2020; Kauffman et al., 2016). Fluorescence and confocal microscopy enable visualization of mitochondrial morphology and spatial localization of MitoSOX or MitoTracker fluorescent probes but are limited by lower throughput, operator-dependent variability, challenges in quantitative standardisation across large sample sets, whereas biochemical assays lack single-cell resolution and often require cell disruption (Mukhopadhyay et al., 2007; Mecca et al., 2025; Cottet-Rousselle et al., 2011). Flow cytometry–based approach described in this protocol offers several significant advantages: (i) it evaluates only the fluorescence signals associated with single cells via small volumes of biological samples and dyes; (ii) it minimizes issues such as fluorescence quenching, light scattering shifts, and interference from no internalized compounds; (iii) it allows the quantification of both the morphological and functional characteristics of intact cells or organelles and facilitates the isolation of highly purified cell populations for mitochondrial studies (Cottet-Rousselle et al., 2011); (iv) the results obtained from the analysis are repeatable and quantifiable, overcoming the operator-dependent limitations of microscopy techniques; and,(v) furthermore, propidium iodide staining permits the discrimination of viable cells, ensuring that analyses are restricted to live cell populations. However, several limitations should be considered when interpreting results. MitoSOX fluorescence may undergo non-specific oxidation. Generating fluorescent products that may not strictly reflect mitochondrial superoxide. In particular, oxidised MitoSOX derivatives can bind nucleic acids, which can markedly amplify fluorescence and alter its apparent intracellular distribution (e.g., increased nuclear/mtDNA-associated signal). Importantly, ROS-inducing treatments may also trigger cell death or early membrane damage, which can confound MitoSOX measurements by increasing dye uptake/retention and by promoting non-specific oxidation and nucleic-acid–associated fluorescence. Therefore, rigorous viability assessment (e.g., exclusion of PI-positive cells) and appropriate positive/negative controls are essential.

MitoTracker Red accumulation depends on mitochondrial membrane potential (Δψm) and may therefore reflect mitochondrial depolarization/hyperpolarization rather than true differences in mitochondrial mass or abundance. Sample processing steps, including enzymatic dissociation (e.g., trypsinisation) and magnetic enrichment, may affect mitochondrial physiology and introduce dye loading variability. In addition, flow cytometry provides quantitative fluorescence measurements without spatial information on mitochondrial morphology.

### 1.2 Development of the protocol

This protocol was developed to enable robust and standardised quantitative assessment of mitochondrial superoxide production and mitochondrial mass in live cancer cells and clinical samples using flow cytometry, adapting fluorescent probes originally designed for fluorescence microscopy to high-throughput single-cell analysis (Cottet-Rousselle et al., 2011; Stryhn et al., 2020; Monteiro et al., 2020). Previous studies have demonstrated the use of MitoSOX-based probes for detecting mitochondrial ROS and MitoTracker dyes for visualising mitochondrial mass in live cells. However, application of these probes in flow cytometry has been limited by variability in staining conditions, probe oxidation, cell handling procedures, flow cytometry gating, and data analysis pipelines.

The present protocol is a standardised, quantitative, and reproducible framework for analysing single-cell mitochondrial profiling providing MSG and MTR probes concentration, incubation time, cell density, washing procedures and acquisition parameters. Standardised gating strategies, fluorescence normalisation methods and quality-control criteria were implemented to improve reproducibility across experiments and laboratories. The protocol also integrates viability discrimination and experimental controls to reduce confounding effects arising from cell death or nonspecific probe accumulation.

To further support protocol reproducibility and applicability independently of the previously published biological study (Mecca et al., 2025), representative validation datasets from multiple independent experiments performed in both immortalised multiple myeloma (MM) and chronic lymphocytic leukaemia (CLL), as well as primary CD138^+^ patient-derived MM cells, are included in the present manuscript. Although developed for cancer models, the workflow can be adapted to additional cell types (e.g., patient-derived cells from different clinical samples) and experimental systems to simultaneously study the mitochondrial superoxide production and mitochondrial mass in live cells by flow cytometry.

Importantly, the use of proper positive controls (e.g., rotenone (Ochi et al., 2016), antimycin A, doxorubicin, high glucose), unstained control, vehicle control (e.g., DMSO, PBS), and viability control (e.g., propidium iodide) to validate mitochondrial superoxide generation with MitoSOX and mitochondrial mass with MitoTracker is very important.

This new dual-dye strategy provides a valuable opportunity to assess multiple mitochondrial parameters in living cells, offering deeper insights into mitochondrial dynamics and their response to experimental conditions.

### 1.3 Overview of the procedure

The protocol consists of sequential steps for isolation of primary CD138^+^ cells, preparation of biological samples, probe staining and flow cytometric analysis.

**FIGURE 2:**
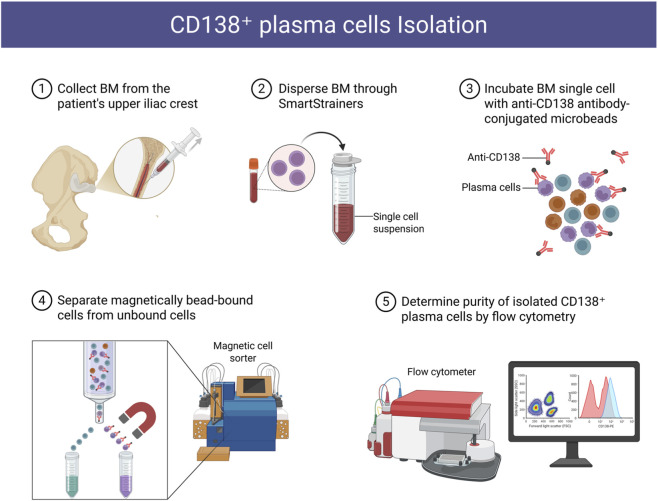
Isolation of CD138^+^ plasma cells from multiple myeloma bone marrow. Workflow for magnetic enrichment of CD138^+^ plasma cells. Bone marrow aspirates are processed to obtain a single-cell suspension, incubated with anti-CD138 magnetic microbeads, separated by magnetic sorting and analysed by flow cytometry to assess purity before downstream applications.

Isolation of CD138^+^ plasma cells (Procedure 3.1, steps 1–15). Primary CD138^+^ plasma cells from multiple myeloma patients are magnetically separated and assessed for purity and viability (Figure 2).

Cancer cell preparation (Procedure 3.2, steps 1–9). MM and CLL cancer cell lines or primary CD138^+^ plasma cells are cultured, treated under defined experimental conditions and harvested at standardised cell densities.

Probe staining (Procedures 3.3–3.5, steps 1–4). Cells are incubated with MitoSOX Green to detect mitochondrial superoxide production and/or MitoTracker Red to assess mitochondrial probe accumulation. Dual staining enables simultaneous measurement of both parameters.

Flow cytometry acquisition and gating (Procedure 3.6, steps 1–6). Cells are stained with a viability dye, followed by sequential gating to exclude debris, doublets and non-viable cells. Fluorescence signals are acquired using standardised instrument settings.

Data analysis (Paragraphs 4–5). Fluorescence intensity values are normalised to controls and expressed as mean or median fluorescence intensity. Integrated indices combining mitochondrial superoxide and mitochondrial mass may be calculated. Reproducibility and quality control are assessed using the coefficient of variation.

**FIGURE 3:**
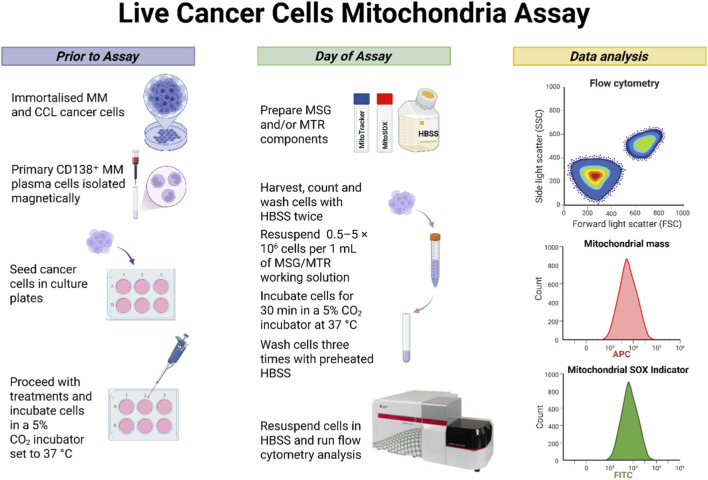
Workflow for mitochondrial functional analysis in live MM and CCL cancer cells and primary CD138^+^ plasma cells. Overview of the experimental procedure for assessment of mitochondrial superoxide production and mitochondrial mass by flow cytometry. Prior to the assay, CD138^+^ plasma cells, isolated from multiple myeloma samples, and immortalised MM and CCL cancer cells are seeded and treated under defined conditions. On the day of the assay, cells are harvested, washed and incubated with MitoSOX Green (MSG) and/or MitoTracker Red (MTR) in HBSS, followed by washing and flow cytometry acquisition. Data analysis includes sequential gating of cell populations and quantification of mitochondrial mass and mitochondrial superoxide fluorescence signals. Created in BioRender by Mecca, M (2026).

A schematic workflow is shown in Figure 3.

### 1.4 Applications

This protocol provides a standardised and quantitative assessment of mitochondrial superoxide production and mitochondrial probe accumulation in viable single cells, providing a functional readout of mitochondrial redox activity and mitochondrial mass in intact cellular systems. The workflow is particularly suited for studies requiring reproducible population-level and single-cell measurements of mitochondrial function under defined experimental conditions.

Proven applications. The method has been validated in haematological cancer models, including multiple myeloma and chronic lymphocytic leukaemia cell lines, as well as primary CD138^+^ plasma cells, where it has been used to characterise mitochondrial oxidative stress and mitochondrial mass following pharmacological or metabolic perturbations (Mecca et al., 2025). These applications demonstrated the utility of the protocol for detecting treatment-induced changes in mitochondrial superoxide generation and mitochondrial functional state in heterogeneous cancer cell populations. The workflow has also been applied to evaluate mitochondrial responses to respiratory chain inhibition and oxidative stress, supporting functional studies of mitochondrial metabolism, redox regulation and metabolic adaptation in tumour cells (Maharjan et al., 2014; Wang et al., 2018; Giddings et al., 2021).

Potential applications. The protocol can be extended to other cancer types, non-transformed cells, patient-derived cells, and clinical samples to investigate mitochondrial alterations associated to disease-relevant processes, such as metabolic reprogramming, oxidative stress responses and mitochondrial dysfunction. Because the method enables functional analysis in intact cells, it can be used to study how mitochondrial activity integrates with cellular signalling pathways and to evaluate the effects of pharmacological agents targeting mitochondrial metabolism or redox balance, including assessment of treatment-induced mitochondrial responses and toxicity. The workflow is compatible with multiparametric flow cytometry and can be combined with surface marker staining to analyse mitochondrial properties within defined cellular subsets or heterogeneous populations. In translational settings, the procedure may also be adapted for functional profiling of patient-derived samples, for example, to investigate mitochondrial dysfunction or treatment response, although such applications require further validation.

### 1.5 Experimental design

To ensure reproducible quantification of mitochondrial fluorescence signals, staining conditions, acquisition parameters and statistical analysis procedures must be predefined and applied consistently across experiments. Because probe uptake, oxidation and mitochondrial membrane potential depend on cellular physiology, experimental parameters should be defined and validated for each biological system before large-scale experiments. Furthermore, appropriate controls must be included for accurate interpretation of fluorescence signals, including unstained and vehicle-treated controls to define baseline fluorescence; positive controls inducing mitochondrial ROS or mitochondrial perturbation; single-colour controls for compensation; fluorescence-minus-one controls where applicable; and viability staining to exclude non-viable cells.

Experimental conditions influencing mitochondrial function must be standardised at the stage of sample preparation (Procedure 3.1–3.2). Cell type, culture density, growth phase and treatment conditions directly affect mitochondrial activity and probe accumulation and should therefore be kept consistent across experimental groups. Cells should be analysed at comparable density and in the logarithmic growth phase, maintaining the recommended concentration at staining (0.5–5 × 10^6^ cells ml^-1^). For primary CD138^+^ cells, viability (>85%) and purity (>90%) are recommended prior to staining; samples should be processed rapidly after collection, and excessive mechanical stress or prolonged handling should be avoided. Under optimised processing conditions, post-isolation viability of primary CD138^+^ plasma cells was typically maintained between 85% and 95%, allowing reliable downstream mitochondrial staining and flow cytometry acquisition. Viability assessment was systematically performed before staining and immediately prior to acquisition to minimise artefacts associated with compromised membrane integrity or nonspecific probe accumulation. Treatment duration and concentration should be optimised in advance, as mitochondrial responses are time dependent. When cryopreserved samples are analysed, all specimens should undergo identical thawing, recovery, and staining procedures to minimize variability associated with freeze–thaw-induced mitochondrial stress.

Probe concentration and incubation parameters (Procedure 3.3–3.5) should be empirically optimised for each cell type to maximise signal-to-noise ratio while preserving cell viability. Excessive probe concentrations may induce cytotoxicity or nonspecific fluorescence, whereas insufficient staining reduces assay sensitivity. Incubation time and temperature should be standardised, and temperature fluctuations during staining or washing minimised. When dual staining is performed, spectral overlap and dye compatibility should be evaluated using single-colour controls to establish compensation settings.

Appropriate controls are essential for correct interpretation of fluorescence measurements (Procedure 3.3–3.6). These include unstained and vehicle-treated samples to determine baseline fluorescence, positive controls that induce mitochondrial ROS or alter mitochondrial function to confirm assay sensitivity, and viability staining to exclude non-viable cells that may exhibit nonspecific probe accumulation. Particular attention was given to exclusion of PI-positive cells during gating analysis, since damaged or dying cells may exhibit altered mitochondrial membrane permeability and non-specific accumulation of fluorescent probes. Control samples should be processed and analysed under identical conditions to experimental samples.

Flow cytometry performance (Procedure 3.6) should be verified daily using calibration standards and requires predefined instrument settings and gating strategies. Detector gain, laser configuration and compensation parameters should be standardised and maintained across all samples, and instrument performance should be verified before acquisition. Sequential gating to exclude debris, doublets and non-viable cells should be applied consistently, and samples should be analysed promptly after staining to minimise variability in fluorescence signals. In our experience, reliable fluorescence quantification can typically be achieved starting from approximately 1–2 × 10^5^ viable cells per condition, although higher input numbers improve robustness for heterogeneous primary samples. For patient-derived CD138^+^ plasma cells, acceptable acquisition quality was routinely obtained despite variability in post-isolation recovery. For primary CD138^+^ plasma cell preparations, lower post-isolation recovery may occur depending on tumour burden and bone marrow cellularity; nevertheless, acquisition of ≥10,000 viable single-cell events was generally achievable under optimised enrichment and staining conditions.

Sample size should be determined based on preliminary experiments evaluating fluorescence variability and expected treatment effects. Experiments should be designed to detect biologically meaningful differences under standardised staining and acquisition conditions. At least three independent biological replicates are recommended, together with technical replicates to assess staining variability. Fluorescence measurements should be normalised to appropriate controls, and variability between replicates monitored using predefined quality criteria. Technical variability should be monitored using the coefficient of variation (CV). Samples should be analysed promptly after staining to minimise fluorescence variability, and experiments with CV >15% or insufficient viability should be repeated.

The protocol can be adapted for different cell types, treatments or multiparametric staining panels; however, probe concentration, incubation conditions and compensation settings may require re-optimisation. Implementation requires access to a flow cytometer equipped with appropriate excitation lasers and detectors, as well as expertise in multiparametric flow cytometry, fluorescence compensation and gating strategies.

### 1.6 Regulatory approvals

This protocol requires the use of primary CD138^+^ cells from multiple myeloma patients for cell culture. Experiments involving primary human samples require institutional ethical approval and informed consent in accordance with local regulations. Processing of human-derived material must comply with institutional biosafety guidelines. The time required to obtain approval varies by institution and may range from several weeks to months.

## 2 Materials and equipment

### 2.1 Biological materials

- MM1S (#CRL-2974, ATCC, Manassas, VA, United States)
- RPMI-8226 (#CCL-155, ATCC, Manassas, VA, United States)
- HG3 (#ACC765, DSMZ, Braunschweig, Germany)
- Patient-derived CD138^+^ multiple myeloma cells

### 2.2 Reagents

- RPMI-1640 Medium (#11–875–093, Life Technologies, United States)
- L–Glutamine (#25030081, Life Technologies, United States)
- Penicillin-Streptomycin Solution (#15140130, Life Technologies, United States)
- Fetal Bovine Serum (#A3160502, Life Technologies, United States)
- Dulbecco’s Phosphate Buffered Saline (#14-190-144, Thermo Fisher Scientific, MA, United States)
- HEPES (#15630080, Thermo Fisher Scientific, MA, United States)
- StraightFrom Whole Blood CD138 MicroBeads (#130-093-062, MiltenyiBiotec, CO, Germany)
- CD138-PE Antibody (#130-127-978, MiltenyiBiotec, CO, Germany)
- CD19-APC Antibody (#130-113-642, MiltenyiBiotec, CO, Germany)
- CD45-FITC Antibody (#130-110-631, MiltenyiBiotec, CO, Germany)
- MACS® Separation Buffer (#130-091-221, MiltenyiBiotec, CO, Germany)
- Trypsin-EDTA (0.25%) (#25200056, Thermo Fisher Scientific, MA, United States)
- Trypan Blue Stain (0.4%) (#15250–061, Life Technologies, United States)
- Dimethyl sulfoxide (DMSO) (#67–68–5, Thermo Fisher Scientific, MA, United States)
- Hanks’ Balanced Salt Solution (HBSS, with Ca^2+^ and Mg^2+^) (#14025–092, Thermo Fisher Scientific, MA, United States)
- Invitrogen MitoSOX Green Mitochondrial Superoxide Indicators (#M36005, Thermo Fisher Scientific, MA, United States)
- Rotenone (#R8875, Sigma Aldrich-Merck, Germany)
- Invitrogen MitoTracker Red CMXRos (#M7512, Thermo Fisher Scientific, MA, United States)
- Propidium Iodide (#P1304MP, Thermo Fisher Scientific, MA, United States)

### 2.3 Equipments

- Cell culture equipment
- 4 and −20 °C refrigerators
- 5 mL Round Bottom Test Tubes (#CLS352052, Sigma Aldrich-Merck, Germany)
- MACS® SmartStrainers (100 µm) (#130-098-463, MiltenyiBiotec, CO, Germany)
- Chill 5 Rack (#130-092-951, MiltenyiBiotec, CO, Germany)
- AutoMACS ProSeparator (#130-092-545, MiltenyiBiotec, CO, Germany)
- Centrifuge (5810R, Eppendorf, Germany)
- Vortex Mixer (F202A0175, VELP Scientifica, Italy)
- Water bath (B3A00035-BTU3, ISCO, Italy)
- Incubator set at 37 °C with 5% CO_2_ (Forma Steri-Cycle, Thermo Fisher Scientific, MA, United States)
- Microscope (Axio Vert A1, Zeiss, Jena, Germany)
- DxFLEX flow cytometer (488 nm blue laser and 638–688 nm red laser, for excitation) (Beckman Coulter, IN, United States)
- Kaluza 2.1 analysis software (Beckman Coulter, IN, United States)
- GraphPad Prism 10 software (San Diego, CA, United States)
- Additional reagents and equipment for counting cells.

### 2.4 Reagent setup

#### 2.4.1 RPMI-1640 medium

Prepare the RPMI-1640 medium by adding 10% (vol/vol) heat-inactivated FBS, 100 U/mL penicillin, 100 mg/mL streptomycin, and 2 mM of L-glutamine. The medium should be stored at 4 °C and used within 1 month.

#### 2.4.2 FACS binding buffer

Hanks’ Balanced Salt Solution containing 5 mM calcium chloride and magnesium chloride.

#### 2.4.3 MitoSOX stock solution

Dissolve the contents (9 μg) of one vial of MSG in 10 μL of dimethyl sulfoxide (DMSO) to make a 1 mM MSG reagent stock solution (Figure 3). Aliquot and store at −20 °C protected from light.

#### 2.4.4 MitoSOX working solution

Dilute 10 μL of 1 mM MSG stock solution to 10 mL of HBSS to make a 1 μM working solution immediately before the experiment (<15 min).

#### 2.4.5 MitoTracker stock solution

Dissolve the contents (50 μg) of one vial of MTR in 94 μL of DMSO to make a 1 mM MTR reagent stock solution (Figure 3). Aliquot and store at −20 °C protected from light.

#### 2.4.6 MitoTracker working solution

Dilute 5 μL of 1 mM MTR stock solution to 10 mL of HBSS to make a 500 nM working solution immediately before the experiment (<15 min).

**⚠** CRITICAL STEP*:* MitoSOX and MitoTracker reagents should be stored at −20 °C and protected from light. They are also easily oxidized, so avoid contact with air.

#### 2.4.7 Rotenone stock solution

Dissolve 5 mg of rotenone in 127 μL of DMSO to make a 100 mM rotenone reagent stock solution. Aliquot and store at −20 °C protected from light.

#### 2.4.8 Rotenone working solution

Dilute 1 μL of 100 mM Rotenone stock solution to 10 mL of PBS to make a 1 mM working solution immediately before the experiment (<15 min).

#### 2.4.9 Media and buffer pre-warming

Place all media or buffers in a 37 °C incubator for preheating for at least half an hour.

**⚠** CRITICAL STEP: Temperature variations may stimulate cells, leading to erroneous mROS and active mitochondria measurement results.

⚠ CRITICAL STEP: All procedures should be carried out at room temperature in a sterile environment unless otherwise specified.

## 3 Methods

This step-by-step protocol consists of the following steps: isolation of CD138^+^ plasma cells, cancer cell seeding and drug treatment on the day prior to the assay, cell harvesting, MSG and MTR staining, and flow cytometric analysis on the day of the test (Figure 3). All steps have been optimised to assess mitochondrial superoxide in live primary or immortalized CCL and MM cancer cells (Mecca et al., 2025).

All procedures described below were developed using a DxFLEX flow cytometer instrument (Beckman Coulter, IN, United States), but they can also be performed on other instruments with the same reagents and compatible acquisition settings. To improve run reliability, at least three replicates per group are recommended. Moreover, consistent results across independent experiments are essential to confirm the findings.

### 3.1 Isolation of CD138^+^ plasma cells from multiple myeloma patients

Timing 3–5 h.

Bone marrow (BM) aspirates or peripheral blood samples are obtained from patients with multiple myeloma following institutional ethical approval and informed consent (Mecca et al., 2025). Samples are processed within 4 h of collection to preserve cell viability. Under these standardised handling conditions, primary samples generally retained high viability throughout the enrichment procedure, supporting subsequent live-cell mitochondrial analyses.

#### 3.1.1 Preparation of mononuclear cells

1. Collect up to 2–10 mL of BM from the upper iliac crest or the sternum using an aspiration needle following informed consent and ethical approval (Figure 2, step 1).
2. Dilute BM aspirate 1:1 with sterile HEPES-buffered cell culture medium.
3. Pass cells through MACS SmartStrainers (100 µm) to remove bone fragments or cell clumps. Wet the filter with the separation buffer before use (Figure 2, step 2).
4. Centrifuge at 445 *g* for 10 min at room temperature without brake.
5. Resuspend the cell pellet in separation buffer to the original volume used in step 1 and determine cell number and viability using trypan blue exclusion. Processing of clinically relevant bone marrow specimens generally yielded sufficient viable CD138^+^ plasma cells to perform mitochondrial staining and flow cytometry analysis without requiring large sample volumes.

**⚠** CRITICAL STEP: Avoid excessive centrifugation or harsh pipetting, which reduces plasma cell viability.


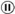
 PAUSE POINT: Isolated CD138^+^ plasma cells may be maintained in complete culture medium at 37 °C and 5% CO_2_ for up to 24 h prior to downstream experiments. Alternatively, cells can be cryopreserved; however, freeze–thaw procedures may affect mitochondrial membrane potential, oxidative status, and fluorescent probe accumulation. Therefore, fresh and cryopreserved samples should be analysed separately whenever possible and processed under standardized recovery conditions. Following thawing, cells should be allowed to recover in complete medium for at least 1 h before staining to reduce transient mitochondrial stress responses. Matched fresh controls are strongly recommended whenever cryopreserved samples are included in comparative analyses.

#### 3.1.2 Magnetic enrichment of CD138^+^ cells

1. Add 50 μL StraightFrom Whole Blood CD138 MicroBeads per 1 mL of BM (Figure 2, step 3).
2. Mix well and incubate for 15 min at 4 °C.
3. Wash cells with 2–5 mL separation buffer per 1 mL of BM and centrifuge at 445 *g* for 10 min at room temperature without brake.
4. Aspirate supernatant carefully and resuspend cells in 500 μL buffer and apply to a magnetic separation column placed in a magnetic field.
5. Place sample and collection tubes into the following Chill Rack positions: position A = sample, position B = negative fraction, position C = positive fraction.
6. Choose program sequence Posselwb/Rinse (repeated magnetic separation) to the AutoMACS ProSeparator (Figure 2, step 4).
7. Collect the flow-through containing CD138^-^ cells.
8. Wash the column three times with separation buffer at room temperature.
9. Remove the column from the magnet and elute CD138^+^ cells by flushing with buffer using the plunger.

**⚠** CRITICAL STEP: Pre-wet columns and perform recommended washing steps to ensure high purity.

#### 3.1.3 Assessment of purity and viability

1. Determine purity of isolated CD138^+^ plasma cells by flow cytometry using antibodies against CD138-PE, CD19-APC, and CD45-FITC (Figure 2, step 5).

⚠ CRITICAL STEP: Use only samples with ≥90% CD138^+^ cells and viability >85% for downstream applications. If viability is less than 85%, repeat isolation.

TROUBLESHOOTING: Low purity may result from cell clumping or insufficient washing; include DNase I (50 U ml^-1^) if necessary.

### 3.2 Cancer cells preparation

Timing 1–24 h.

1. Seed 0.5 × 10^6^ cells mL^-1^ cancer cells in culture plates or flasks to assess changes in mitochondrial superoxide/ROS in live cancer cells, achieving 0.5–5 × 10^6^ cells mL^-1^ at staining (Figure 3).
2. Apply experimental treatments on cancer cells (e.g., drug treatments, silencing, fasting, *etc.*) whose mitochondrial effect will be evaluated.

TROUBLESHOOTING: Ensures the minimum cell density (0.5–5 × 10^6^ cells per mL) required on the day of the assay.

TROUBLESHOOTING: If they have an immediate effect, proceed on the same day as the analysis.

3. Prepare a positive control by treating cancer cells with ROS-activating inhibitors such as rotenone (0.1–10 μM) (Ochi et al., 2016).
4. Incubate cell culture plates or flasks in a 5% CO_2_ incubator at 37 °C overnight (Figure 3).
5. Shake gently the culture plates or flasks and remove the culture cell medium to discard the dead cells or ageing cells with poor adherence.
6. Add preheated trypsin-EDTA (0.25%) to the cells and hold at approximately 18 °C–25 °C until they shrunk and round (after almost 5 min).

TROUBLESHOOTING: If the cells are in suspension, harvest them and proceed to step 8.

7. Neutralise trypsin-EDTA by adding preheated complete medium (twice the volume of trypsin-EDTA).
8. Harvest and count the trypan blue/cell mixture under a microscope.
9. Centrifuge cells at 400 × g for 5 min and wash with HBSS twice at room temperature.

### 3.3 MitoSOX green staining

Timing 45–60 min.

1. Resuspend cancer cells to a concentration of 0.5–5 × 10^6^ cells per 1 mL of 1 μM MSG working solution and shake gently (Figure 3).

⚠ CRITICAL STEP: It is important to use the same cell number for all samples if you want to compare their results.

⚠ CRITICAL STEP: The working solution is stable for 1 day and should be protected from light and stored at 4 °C.

TROUBLESHOOTING: Although the recommended concentration of the working solution is 1 μM, it can be optimised from 0.5 μM to 5 μM depending on the cell type to maximize the signal-to-noise ratio and minimize cellular toxicity. The concentration of the MSG should not exceed 5 μM, as concentrations above 5 μM may produce cytotoxic effects (e.g., altered mitochondrial morphology and redistribution of fluorescence in the nucleus and cytosol).

2. Prepare a negative control by adding the same amount of PBS or vehicle control instead of MSG.
3. Incubate cells for 30 min in a 5% CO_2_ incubator at 37 °C protected from light.

**⚠** CRITICAL STEP: The incubation of cells with preheated MSG should last 15 min–30 min at 37 °C; otherwise, the degree of staining will not be sufficient.

4. Wash cells gently three times with preheated HBSS and centrifuge at 400 *g* for 3 min at room temperature.

### 3.4 MitoTracker red staining

Timing 45–60 min.

1. Resuspend cancer cells to a concentration of 0.5–5 × 10^6^ cells per 1 mL of 500 nM MTR working solution and shake gently (Figure 3).

**⚠** CRITICAL STEP: It is important to use the same cell number for all samples if you want to compare their results.

⚠ CRITICAL STEP: The working solution is stable for 1 day and should be protected from light and stored at 4 °C.

TROUBLESHOOTING: Although the recommended concentration of the working solution is 500 nM, it can be optimised from 25 nM to 500 nM depending on the cell type to maximize the signal-to-noise ratio and minimize cellular toxicity. The concentration of the MTR should not exceed 500 nM, as concentrations above 5 μM may produce cytotoxic effects (e.g., altered mitochondrial morphology and redistribution of fluorescence in the nucleus and cytosol).

2. Prepare a negative control by adding the same amount of PBS or vehicle control instead of MTR.
3. Incubate cells for 30 min in a 5% CO_2_ incubator at 37 °C protected from light.

**⚠** CRITICAL STEP: The incubation of cells with preheated MTR should last 15 min–45 min at 37 °C; otherwise, the degree of staining will not be sufficient.

4. Wash cells gently three times with prewarmed HBSS and centrifuge at 400 *g* for 3 min at room temperature.

### 3.5 MSG and MTR dual staining

Timing 45–60 min.

**FIGURE 4:**
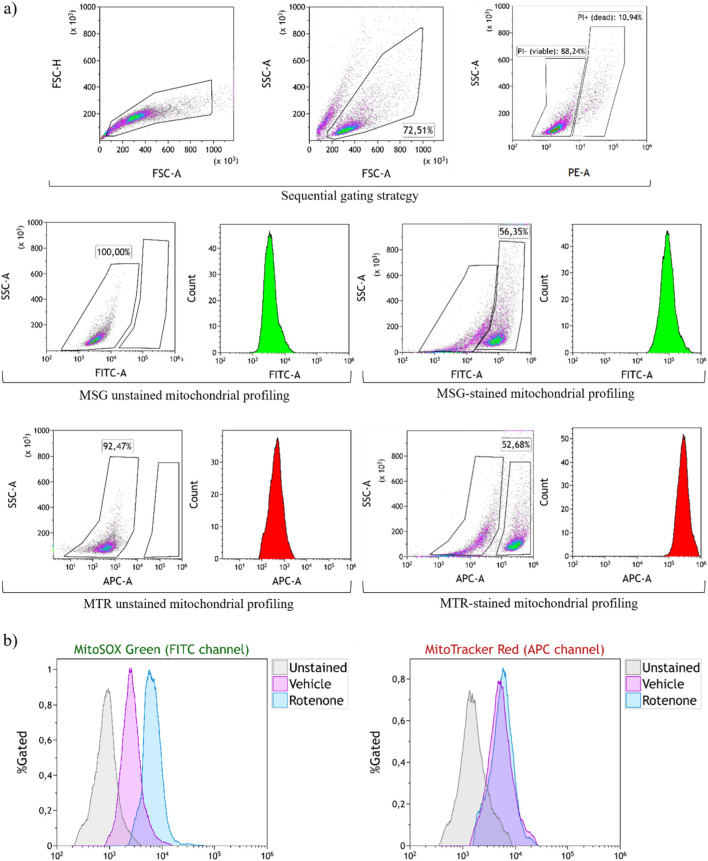
Representative flow cytometry gating strategy and mitochondrial functional profiling using MSG and MTR. **(a)** Sequential gating strategy including debris exclusion (FSC-A vs. SSC-A), singlet discrimination (FSC-H vs. FSC-A), viability gating by propidium iodide (PI) exclusion (PE channel), and median fluorescence intensity (MFI) of MSG (FITC channel) and MTR (APC channel) signals using unstained controls and standardised acquisition settings. **(b)** Overlay histograms of unstained, vehicle-treated, and rotenone-treated samples. MSG fluorescence reflects mitochondrial superoxide production, whereas MTR fluorescence reflects mitochondrial probe accumulation associated with mitochondrial mass and membrane potential. Data are representative of independent experiments performed under standardised acquisition conditions.

MTR is well suited for multicolour labelling experiments because its red fluorescence is well resolved from the green fluorescence of other probes. Therefore, owing to the slight difference in excitation and emission wavelengths between red fluorescence MTR and MSG, it is possible to use them simultaneously in the same assay (Figure 3). This allows us to obtain information on both the mitochondrial superoxide level and the mitochondrial mass of each sample in a single analysis (Mecca et al., 2025). Compared with the two procedures described above, only the following is necessary:

1. Incubate cells in a combined solution containing both 1 μM MSG and 500 nM MTR.
2. Select both the FITC and APC filters on the flow cytometer (Figure 4).

### 3.6 Flow cytometry acquisition and gating

Flow cytometry data were acquired using a DxFLEX Flow Cytometer (Beckman Coulter) equipped with 488 nm and 638 nm excitation lasers and configured with standard bandpass filters (baseline voltage range 100 V–240 V ± 10%, 50 Hz–60 Hz ± 1 Hz). FITC fluorescence was detected using a 525/40 bandpass filter following excitation with the 488 nm blue laser, whereas APC fluorescence was detected using a 660/10 bandpass filter following excitation with the 638 nm red laser. Instrument performance is verified daily using calibration beads according to the manufacturer’s recommendations. A standardized gating template is applied across all experiments to ensure reproducibility.

Timing 1–2 h.

1. Stain the cells with a viability dye, such as 50 μg/mL propidium iodide, and incubate for 15 min at room temperature (25 °C) before analysis.

⚠ CRITICAL STEP: Keep samples cold during acquisition on the cytometer. Run samples as soon as possible, at the latest within 30 min of the addition of dyes.

2. Prepare the cells for flow cytometry analysis by transferring the cell suspension into FACS tubes (Figure 3).

⚠ CRITICAL STEP: Ensure that all flow cytometry equipment is set up in advance, since cells should be examined within 2 h of staining.

3. Sequential gating should be performed as follows:
  - Debris exclusion: identify cell populations based on forward scatter (FSC-A) versus side scatter (SSC-A) properties. Exclude debris and non-cellular events outside the main cell population.
  - Doublet discrimination: select singlet cells and exclude doublets using FSC-H versus FSC-A (or FSC-W versus FSC-A) gating.
  - Viability gating: exclude non-viable cells based on propidium iodide or equivalent viability dye staining. Only PI-negative viable single cells were included in downstream fluorescence quantification analyses.
  - Threshold determination: define fluorescence-positive populations and thresholds using unstained controls and fluorescence-minus-one controls and apply uniformly across samples.
  - Quantification of MSG and MTR fluorescence exclusively within the viable singlet population;
  - Acquire at least 10,000 viable single-cell events per sample. When analysing low-input patient-derived samples, acquisition settings should be optimised to minimise cell loss and maintain stable event rates throughout acquisition.

⚠ CRITICAL STEP: Individual control samples stained with each of the dyes separately are needed for instrument setup.

5. Run samples on the flow cytometer with 488 nm excitation and 525/40 nm emission to measure oxidised MSG; therefore, its expression is quantified in a standard bandpass FITC filter.

TROUBLESHOOTING: It is advisable to analyse the samples from low to high FITC fluorescence.

6. Run samples on the flow cytometer with 638 nm excitation and 660/10 nm emission to measure oxidised MTR; therefore, its expression is quantified in a standard bandpass APC filter.

TROUBLESHOOTING: It is advisable to analyse the samples from low to high APC fluorescence.

7. Export data and perform data analysis. Plot cells for FSC and SSC: cell debris with low FSC and SSC should be excluded from analyses. Then, plot viable cells for propidium iodide, followed by plotting each population as a histogram of mean fluorescence intensity (FITC for MSG and APC for MTR) (Figure 4).

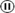
 **PAUSE POINT:** Acquired flow cytometry data can be stored indefinitely for downstream analysis. Processed and compensated data may be analysed at any time using consistent gating strategies.

Troubleshooting advice can be found in Table 1.

**TABLE 1:** Troubleshooting table.

Step	Problem	Possible reason	Solution
CD138^+^ isolation — mononuclear cell preparation	Low plasma cell yield	Poor sample quality or delayed processing	Process samples within 4 h of collection; store samples at 4 °C before processing
	Low cell viability	Excessive centrifugation or harsh pipetting	Use gentle pipetting; centrifuge ≤445 g without brake
CD138^+^ magnetic separation	Low CD138^+^ purity	Cell clumping	Filter cells through 100 μm strainer; add DNase I (50 U ml^-1^) if necessary
	Low CD138^+^ purity	Insufficient washing or incomplete magnetic separation	Pre-wet columns and perform recommended washing steps
	Low recovery of CD138^+^ cells	Incomplete elution from column	Remove column from magnet and flush thoroughly with buffer
CD138^+^ purity assessment	High CD19^+^ or CD45^+^ contamination	Non-specific antibody binding	Include fc-blocking step or increase washing stringency
Cancer cell preparation	Insufficient cell number at assay	Low initial seeding density	Ensure 0.5–5 × 10^6^ cells ml^-1^ at staining
	Poor cell attachment or excessive death	Over-trypsinization or stress	Monitor detachment carefully and neutralize trypsin promptly
	Loss of suspension cells	Incorrect harvesting method	For suspension cells skip trypsinization and harvest directly
MitoSOX staining	Weak fluorescence signal	Low dye concentration or short incubation	Optimize MSG concentration (0.5–5 μM); incubate 15–30 min at 37 °C
	High background fluorescence	Excess dye or insufficient washing	Reduce dye concentration and increase wash steps
	Cytotoxicity or altered mitochondrial morphology	MSG concentration too high (>5 μM)	Reduce dye concentration
	Uneven staining between samples	Different cell numbers or temperature variation	Use identical cell numbers and pre-warmed buffers
	Probe oxidation before use	Exposure to air or light	Prepare working solution fresh and protect from light
MitoTracker staining	Weak mitochondrial signal	Insufficient incubation time or low concentration	Optimize MTR concentration (25–500 nM) and incubation time
	Cytotoxic effects	Excessive MTR concentration	Do not exceed 500 nM working concentration
	Loss of mitochondrial signal	Loss of membrane potential	Reduce experimental stress and verify cell viability
	High background fluorescence	Excess dye or incomplete washing	Increase wash steps and reduce dye concentration
Dual MSG/MTR staining	Signal overlap between channels	Spectral spillover or improper compensation	Perform single-stained controls and adjust compensation matrix
	Reduced staining efficiency	Dye competition or incorrect incubation conditions	Validate dual staining conditions and optimize dye concentrations
Flow cytometry acquisition	High proportion of dead cells	Improper handling or delayed analysis	Analyse cells within 30–120 min after staining and maintain samples on ice
	Weak fluorescence detection	Incorrect detectors gain or laser settings	Optimize detector gain daily using unstained and single-stained controls
	Inconsistent fluorescence between runs	Instrument drift	Perform daily instrument quality control and calibration
	Debris contamination	Inadequate gating or sample preparation	Filter samples and gate FSC/SSC to exclude debris
​	Low event count	Cell loss during washes or low concentration	Increase acquisition time and verify cell concentration
	Carryover between samples	Insufficient fluidics washing	Run sheath fluid wash between samples
	Poor separation of positive/negative populations	Incorrect analysis order	Acquire samples from low to high fluorescence
	Inaccurate compensation between FITC and APC	Improper single-color controls	Prepare compensation controls for each dye
Data analysis	High variability between replicates	Inconsistent staining or cell handling	Standardize staining conditions and use technical replicates
	Misinterpretation of mitochondrial mass	Changes in membrane potential rather than mass	Interpret MTR signal together with viability and experimental context

## 4 Data analysis

Analyse fluorescence intensity data via Kaluza 2.1 analysis software (Figures 4a,b), normalizing to unstained controls. Fluorescence intensity is quantified as either median fluorescence intensity (MFI) (recommended for skewed distributions) or mean fluorescence intensity when distributions are approximately normal. MFI values should be extracted for each gated population.

### 4.1 Normalized fluorescence values

Correct background fluorescence using Equation 1 for unstained or vehicle-treated controls**:**

$$MFI_{corrected}=MFI_{sample}‐MFI_{background}$$ (1)

where:

-
$MFI_{sample}$ = measured fluorescence of stained cells
-
$MFI_{background}$ = fluorescence of unstained control

Normalise the fluorescence signals by applying Equation 2 to the untreated or vehicle-treated control samples to enable comparison across experiments:

$$Normalised\;signal=\frac{MFI_{sample}}{MFI_{control}}$$ (2)

### 4.2 Relative mitochondrial superoxide production

Express relative mitochondrial superoxide levels as normalized MitoSOX fluorescence using Equation 3:

$$Relative\;mitochondrial\;superoxide=\frac{MFI_{MitoSOX,corrected}}{MFI_{control}}$$ (3)

### 4.3 Relative mitochondrial mass

Determine relative mitochondrial mass from normalised MitoTracker fluorescence using Equation 4:

$$Relative\;mitochondrial\;mass=\frac{MFI_{MitoTracker,corrected}}{MFI_{control}}$$ (4)

Because MitoTracker accumulation depends on mitochondrial membrane potential, changes in fluorescence are interpreted within the experimental context.

### 4.4 Mitochondrial functional index (MFIx)

To integrate mitochondrial superoxide production and mitochondrial mass into a single quantitative metric, calculate a mitochondrial functional index (MFIx) by applying Equation 5:

$$MFIx=\frac{MFI_{MitoSOX,corrected}}{MFI_{MitoTracker,corrected}}$$ (5)

This ratio reflects mitochondrial oxidative activity relative to mitochondrial mass and enables comparison of mitochondrial functional states across conditions.

### 4.5 Reproducibility and quality control

Assess signal variability using the coefficient of variation (CV) as defined in Equation 6:

$$CV\left(\%\right)=\frac{Standard\;deviation}{Mean}\times 100$$ (6)

Acceptable technical variation is defined as CV < 15% under standardized conditions. Experiments exceeding this threshold are repeated.

## 5 Statistical analyses

Perform statistical analysis via GraphPad Prism version 10.6 (GraphPad Software, Inc., San Diego, CA, United States) or equivalent software. Report data (mean/median fluorescence) as mean ± standard deviation (SD) for normally distributed data or median with interquartile range for non-normally distributed data from at least three independent experiments performed in triplicate. Assess data distribution using the Shapiro–Wilk normality test. For normally distributed data, perform comparisons between two groups using an unpaired two-tailed Student’s t-test, followed by multiple comparisons using one-way or two-way analysis of variance (ANOVA) with appropriate *post hoc* testing. For non-normally distributed data, apply non-parametric tests (Mann–Whitney U test or Kruskal–Wallis test).

According to the p-value, differences are considered statistically significant when the value of *p < 0.05; **p < 0.01; ***p < 0.001; ****p < 0.0001.

Sample size was determined based on prior studies and preliminary experiments assessing assay variability and expected effect size. A minimum of three independent biological replicates was used to ensure reproducibility under standardized conditions.

## 6 Timing

Procedure 3.1.

Steps 1–5, MC preparation: 30–45 min.

Steps 6–14, CD138^+^ isolation: 2–3 h.

Step 15, CD138^+^ purity and viability: 30–60 min.

Procedure 3.2.

Steps 1-9, Cell seeding: 1 h for evaluation immediately; 24 h for experimental treatments evaluation.

Procedure 3.3.

Steps 1-4, MSG staining: 45–60 min.

Procedure 3.4.

Steps 1-4, MTR staining: 45–60 min.

Procedure 3.5.

Steps 1-2, MSG and MTR dual staining: 45–60 min.

Procedure 3.6.

Steps 1-6, Flow cytometry acquisition: 1–2 h; depending on the number of preparations and events, the process may take up to 2–6 h.

## 7 Anticipated results

Mitochondrial-targeted fluorescent dyes, such as MitoSOX and MitoTracker probes, offer several advantages: they are easy to use, rapidly and selectively target mitochondria, and enable specific detection of superoxide radicals and the mitochondrial mass in live cells. Most studies utilising MitoSOX and MitoTracker have employed fluorescence or confocal microscopy, as these probes were originally designed for visualisation through imaging-based techniques (Liao et al., 2020; Chazotte, 2011; Neikirk et al., 2023; Will et al., 2006). Instead, the present protocol provides a step-by-step standardised flow cytometry workflow for reproducible quantification of mitochondrial superoxide production and mitochondrial mass at single-cell resolution in both live cancer cell lines and primary CD138^+^ patient-derived plasma cells with MSG and MTR probes. A key advantage of the present workflow compared with previously reported mitochondrial flow cytometry assays is the integration of standardised staining conditions, harmonised gating criteria, dual-parameter mitochondrial analysis, and applicability to clinically relevant primary samples. Under optimized conditions, the workflow yields reproducible fluorescence measurements with low technical variability, ensures preservation of cellular function, and enables discrimination of mitochondrial functional states across experimental conditions.

### 7.1 *CD138* ^*+*^ *plasma cell isolation*

Processing of 1 mL of bone marrow aspirate typically yields approximately 1–5 × 10^6^ mononuclear cells, depending on sample cellularity. Magnetic enrichment generally results in CD138^+^ plasma cell populations with ≥90% purity and cell viability exceeding 85%. Lower purity may occur in samples with low tumour burden or extensive cell aggregation. Representative flow cytometry analysis should demonstrate enrichment of CD138^+^ plasma cells with reduced CD19 and CD45 expression (Bansal et al., 2021).

### 7.2 Mitochondrial staining and fluorescence detection

After sequential gating to exclude debris, doublets and non-viable cells, approximately 70%–90% of acquired events generally correspond to viable single cells. Primary CD138^+^ plasma cell preparations typically maintained viability above 85% during staining and acquisition procedures when processed under optimised conditions. Similar viability ranges were observed after short-term culture recovery, supporting the feasibility of the workflow for clinically relevant low-input samples. Acquisition of at least 10,000 viable events per sample typically provides sufficient statistical robustness for quantitative comparisons. Even in low-input primary multiple myeloma samples, reliable fluorescence measurements were generally achievable provided that adequate viability and enrichment quality were maintained throughout processing. Although sample recovery varied among patient specimens, optimized magnetic enrichment and standardized staining conditions consistently enabled acquisition of sufficient viable events for downstream mitochondrial analysis.

Fluorescence intensity is quantified as median fluorescence intensity (MFI), which is recommended because fluorescence distributions are often skewed, or as mean fluorescence intensity when distributions are approximately normal. Relative mitochondrial superoxide production and mitochondrial mass are therefore expressed as normalised MSG and MTR fluorescence, respectively.

In a well-performing experiment, fluorescence signals show low baseline intensity in unstained and vehicle-treated controls and clear population shifts following mitochondrial perturbation. For example, in multiple myeloma cell lines analysed in three independent experiments, unstained cells typically show minimal fluorescence (MSG MFI ≈120 ± 10 arbitrary units (a.u.); MTR MFI ≈95 ± 8 a. u.), whereas vehicle-treated control cells show stable basal fluorescence (MSG MFI ≈480 ± 35 a. u.; MTR MFI ≈620 ± 40 a. u.) with high viability (>90%) (Mecca et al., 2025). Treatment with a mitochondrial stressor such as rotenone (1 μM) typically induces a marked rightward shift of the MSG fluorescence histogram, corresponding to a 2–5-fold increase in normalised MSG signal (for example, MSG MFI ≈1,820 ± 120 a. u., relative MSG ≈3.8 a. u.). Under these conditions, MTR fluorescence may remain stable or increase modestly (for example, MTR MFI ≈710 ± 55 a. u., relative MTR ≈1.15 a. u.), resulting in an increased mitochondrial functional index. Propidium iodide positivity is typically below 10%–15% (Riccardi and Nicoletti, 2006). Histogram overlays show clear separation between unstained, control and treated populations, with treated cells displaying a distinct rightward shift in the FITC channel (MSG) and stable or slightly shifted distributions in the APC channel (MTR). By contrast, problematic experiments are characterised by poor signal resolution or inconsistent fluorescence measurements. Elevated baseline fluorescence in unstained controls, broad or multimodal distributions and weak separation between populations are commonly observed when probe oxidation occurs before staining, dye concentration is excessive or washing steps are insufficient. Histograms from problematic experiments typically show broad peaks without clear maxima and overlapping fluorescence distributions between conditions. Simultaneous loss of MSG and MTR signals frequently indicates mitochondrial depolarisation or extensive cellular damage, whereas high background fluorescence generally reflects nonspecific probe accumulation or incomplete removal of excess dye. Similar transient fluorescence alterations may occasionally be observed immediately after thawing cryopreserved samples and should therefore be interpreted within the context of sample handling conditions.

MSG fluorescence reflects mitochondrial superoxide production through oxidation-dependent probe activation; therefore, an increased MSG signal indicates elevated mitochondrial oxidative activity. However, MitoSOX oxidation is not entirely specific for superoxide and may also be influenced by nonspecific oxidation under conditions of severe oxidative stress or probe overaccumulation. Therefore, fluorescence signals should always be interpreted within the broader biological and experimental context.

MTR fluorescence reflects probe accumulation driven primarily by mitochondrial membrane potential and is commonly interpreted as an indicator of mitochondrial mass, although changes may also reflect alterations in mitochondrial polarisation or functional state. Because MitoTracker accumulation is partially dependent on mitochondrial membrane potential, decreases in fluorescence intensity may reflect mitochondrial depolarisation rather than absolute reductions in mitochondrial mass. Interpretation of experimental results is therefore strengthened by simultaneous analysis of both parameters. Increased MSG together with stable or increased MTR typically indicates enhanced mitochondrial oxidative metabolism, whereas increased MSG accompanied by reduced MTR suggests mitochondrial dysfunction or early apoptotic processes. An increase in MTR without a corresponding change in MSG may reflect mitochondrial biogenesis or adaptive metabolic responses. Notably, resilient mitochondria tend to increase MTR signals for an extended period as part of adaptive survival mechanisms. In contrast, mitochondria, which are more susceptible to damage, show a rapid decline in MTR signals, which is indicative of mitochondrial dysfunction and the early onset of apoptosis (Stryhn et al., 2020; Monteiro et al., 2020).

Although the combined use of MSG and MTR enables sensitive assessment of mitochondrial functional states, interpretation of fluorescence changes should consider potential probe-related limitations, including nonspecific oxidation, mitochondrial depolarisation, variable dye retention, and stress-induced alterations in mitochondrial physiology.

### 7.3 Data quality and analysis

Instrument performance should produce stable fluorescence signals with low background noise and consistent signal intensity across replicates. Fluorescence distributions in successful experiments are generally unimodal with narrow peak widths and low technical variability. Across independent experiments performed in MM1S, RPMI-8226, HG3 and primary CD138^+^ plasma cells, assay reproducibility remained within predefined acceptance criteria, with technical CV values generally below 10%–15% and viability consistently maintained above 85% under optimized staining conditions; higher variability indicates suboptimal staining, inconsistent cell handling or instrument instability. Importantly, exclusion of non-viable PI-positive events significantly reduced fluorescence variability and improved reproducibility across independent experiments.

Cancer cell lines generally exhibit highly reproducible fluorescence profiles across independent experiments. Primary patient samples display greater variability due to biological heterogeneity, differences in mitochondrial activity and variability in sample processing. Additional variability may be observed in cryopreserved specimens because freeze–thaw stress can transiently influence mitochondrial membrane polarization and reactive oxygen species production. Accordingly, direct comparisons between fresh and cryopreserved samples should be performed cautiously and preferably using matched processing conditions and appropriate experimental controls.

Overall, the protocol enables a robust, reproducible and scalable framework for the quantitative assessment of mitochondrial function, enabling sensitive and standardised comparisons analysis across experimental conditions and diverse biological samples.

## Data Availability

The original contributions presented in the study are included in the article/supplementary material, further inquiries can be directed to the corresponding authors.

## Glossary

ANOVA: Analysis of variance
APC: Allophycocyanin
ATP: Adenosine triphosphate
a.u.: Arbitrary units
BM: Bone marrow
°C: Degree Celsius
CD138: Cluster of differentiation 138
CLL: Chronic lymphocytic leukaemia
**CO**_**2**_: Carbon dioxide
CV: Coefficient of variation
DMSO: Dimethyl sulfoxide
EDTA: Ethylenediaminetetraacetic acid
FAD: Flavin-adenine dinucleotide
**FADH**_**2**_: Reduced flavin-adenine dinucleotide
FITC: Fluorescein 5(6)-isothiocyanate
FSC: Forward scatter
GSH: Glutathione
GSSG: Glutathione disulfide
**H**_**2**_ **O**_**2**_: Hydrogen peroxide radicals
HBSS: Hanks’ Balanced Salt Solution
MFI: Median fluorescence intensity
MM: Multiple myeloma
MSG: MitoSOX Green
MTR: MitoTracker Red
**NAD** ^**+**^: **β-nico**tinamide-adenine dinucleotide
NADH: Reduced β-nicotinamide-adenine dinucleotide
**NADP** ^**+**^: β-nicotinamide-adenine dinucleotide phosphate
NADPH: Reduced β-nicotinamide-adenine dinucleotide phosphate
**O**_**2**_^**−**^: Superoxide radicals
OH: Hydroxyl radicals
RNS: reactive nitrogen species
ROS: reactive oxygen species
SD: standard deviation
SSC: side scatter
Δψm: Mitochondrial membrane potential
